# Physics-informed self-supervised diagnosis of rotating machinery using latent ODEs and transformer encoders

**DOI:** 10.1371/journal.pone.0339239

**Published:** 2026-02-02

**Authors:** Md Al Amin, Mohammad Shafat Ahsan, Jannatul Maua, Mumtahina Ahmed, Kamruddin Nur

**Affiliations:** 1 MS in Information Technology, St. Francis College, Brooklyn, New York, United States of America; 2 MSc in IT Project Management, School of Professional Studies, Clark University, Worcester, Massachusetts, United States of America; 3 Department of Computer Science and Engineering, Bangladesh University of Business and Technology, Dhaka, Bangladesh; 4 Department of Computer Science, American International University-Bangladesh, Dhaka, Bangladesh; The Hong Kong Polytechnic University, CHINA

## Abstract

This paper proposes a novel Physics-Informed Self-Supervised Diagnosis (PI-SSD) framework for rotating machinery fault detection, combining physical modeling, self-supervised representation learning, and uncertainty-aware classification. The architecture integrates a multi-resolution convolutional encoder, a windowed Transformer for temporal context modeling, and a latent neural ordinary differential equation (ODE) module that embeds mechanical priors, such as Jeffcott rotor dynamics, directly into the learning process. A masked segment reconstruction objective enables self-supervised pretraining using unlabeled healthy signals, while an evidential classifier head produces fault probabilities with calibrated uncertainty. We evaluate PI-SSD on two publicly available datasets, the NASA PHM’09 Gearbox dataset and the Aalto Rotor dataset, covering 6 fault types and over 5,500 multichannel vibration recordings. Compared to seven strong baselines, PI-SSD achieves the highest Macro-F1 score (0.91) and lowest Expected Calibration Error (ECE = 0.022) on the NASA dataset, while maintaining strong domain transfer performance on Aalto (Macro-F1 = 0.81, PR-MSE = 0.067) without fine-tuning. Ablation studies confirm the contribution of each component, and physics consistency analysis demonstrates low violation rates under varying speeds. These results highlight the potential of embedding physics knowledge into self-supervised neural systems for robust, interpretable, and transferable fault diagnosis in industrial applications.

## 1 Introduction

Gearboxes, turbines, and motors are examples of rotating machinery that are essential to the proper operation of industries. Unexpected failures of these systems can result in expensive downtime, production losses, and even safety hazards. For predictive maintenance and to guarantee consistent performance in the manufacturing, energy, and transportation sectors, early and precise problem identification is crucial. Vibration-based condition monitoring has emerged as one of the best methods for automatically evaluating the health of these equipment due to the increasing usage of sensors and the development of Industrial Internet of Things (IIoT) technology.

Traditional supervised approaches such as convolutional neural networks (CNNs) [1,2] and recurrent networks (RNNs) [3–5] have shown promise in learning patterns from labeled vibration signals, but they often operate as black-box classifiers with little regard for underlying physical laws. More recently, physics-informed neural networks (PINNs) have sought to incorporate governing equations into learning models [6–8]. While this enhances physical plausibility, most PINNs are designed for direct numerical regression and do not support classification, sequence modeling, or self-supervised learning. Separately, self-supervised learning (SSL) has emerged as a powerful tool for pretraining models using unlabeled data [9–11]. Yet, existing SSL methods are largely physics-agnostic and may learn spurious correlations if not constrained by domain knowledge. There remains a critical need for frameworks that integrate physical structure with the flexibility of modern representation learning to enable scalable, interpretable, and generalizable machinery diagnostics.

In this paper, we propose a unified framework called Physics-Informed Self-Supervised Diagnosis (PI-SSD), which addresses these challenges by embedding physical consistency into a self-supervised temporal encoder-decoder architecture. PI-SSD combines multiple key innovations: it uses a masked segment reconstruction task to learn from unlabeled healthy signals, incorporates a latent ordinary differential equation (ODE) module to enforce mechanical constraints (Jeffcott rotor dynamics), models temporal structure via a windowed Transformer, and outputs calibrated uncertainty through a Dirichlet evidential classifier. This design allows the model to learn robust latent dynamics that align with both labeled and unlabeled vibration data while respecting physical principles governing rotating machinery.

We validate PI-SSD on two challenging datasets: the NASA PHM’09 Gearbox dataset, which includes labeled fault modes under varying speed and load conditions, and the Aalto Rotor dataset, which consists of clean healthy behavior across multiple operating frequencies and stiffness configurations. Our results demonstrate that PI-SSD achieves superior performance across classification, calibration, and physics consistency metrics and is capable of zero-shot domain transfer without fine-tuning. Moreover, ablation and sensitivity analyses confirm the effectiveness and robustness of each architectural component.

This work presents a novel methodology that combines these approaches, which is in contrast to previous efforts that either apply current self-supervised or physics-informed techniques to particular datasets. The proposed Physics-Informed Self-Supervised Diagnosis (PI-SSD) framework is not a direct application of previous methods; it represents a new class of hybrid models that jointly optimize masked reconstruction, latent physics residuals, and evidential uncertainty within a single Transformer-based architecture. This formulation advances both the methodological frontier of self-supervised learning for time-series data and its application to rotating machinery diagnostics. Accordingly, our revised Introduction and Related Work

sections now emphasize this dual contribution methodological innovation and domain adaptation-to clarify the paper’s original position within the literature. The key contributions of this paper are summarized as follows:

- We introduce **PI-SSD**, an original physics-informed self-supervised framework that unifies three complementary paradigms-latent ODE-based physical regularization, temporal Transformer modeling, and evidential uncertainty-aware classification for vibration-based machinery diagnostics.
- We design a multi-task learning scheme that jointly optimizes masked segment reconstruction, physics residual minimization, and evidential loss, enabling the model to learn physically consistent and uncertainty-calibrated representations from both labeled and unlabeled signals.
- We embed the Jeffcott rotor and gear-mesh differential equations within the training process as differentiable constraints, linking model predictions directly to governing mechanical dynamics.
- We demonstrate that the proposed framework generalizes across domains by pretraining on healthy Aalto Rotor data and transferring to fault-labeled NASA Gearbox data without fine-tuning, achieving state-of-the-art accuracy, calibration, and physical consistency.
- We provide extensive benchmarking, ablation, and sensitivity analyses that quantify the individual impact of each component and verify that PI-SSD offers interpretable, physically grounded, and statistically reliable fault diagnosis.

The remainder of this paper is organized as follows. Sect 2 reviews related work on data-driven fault diagnosis, physics-informed learning, and self-supervised methods. Sect 3 presents the proposed PI-SSD methodology, including data preprocessing, architectural design, training strategy, and loss functions. Sect 4 describes the experimental setup, datasets, and evaluation metrics, quantitative and qualitative results, including benchmark comparisons, ablation analyses, and calibration performance. Sect 5 discusses the broader implications, novelty, and limitations of the work. Finally, Sect 6 concludes the paper and outlines directions for future research.

## 2 Related work

This section reviews prior work across three key domains that intersect in our proposed framework: data-driven fault diagnosis, physics-informed machine learning, and self-supervised representation learning. We highlight limitations in each area that motivate the design of the proposed PI-SSD architecture.

Deep learning has transformed vibration-based fault diagnosis by enabling end-to-end pattern recognition from raw time-series data. Convolutional Neural Networks (CNNs) have been widely applied to detect localized fault signatures in gearboxes and bearings [12]. Recurrent Neural Networks (RNNs), particularly LSTMs, have been used to model temporal dependencies for early fault progression detection [13]. More recently, Transformer-based models have been introduced to capture long-range context and periodic behavior in vibration sequences [14]. However, these approaches typically rely on supervised training with large amounts of labeled fault data, which is expensive and time-consuming to collect. They also lack physical interpretability, leading to black-box predictions that may not generalize across machinery domains or operating regimes. Chen et al. [15] propose a Double-Scale Convolutional Neural Network (DS-CNN) for vibration-based fault diagnosis of railway point machines. Their architecture captures multi-scale temporal features to improve local and global fault representation under supervised learning. Yang et al. [16] develop VSC-ACGAN, an adversarial generative model that addresses data imbalance in bearing fault diagnosis by synthesizing realistic minority-class samples. Both approaches demonstrate high accuracy on domain-specific datasets but rely heavily on labeled data and do not embed physical modeling or self-supervised objectives. In contrast, our PI-SSD framework integrates latent ODE-based physics constraints and self-supervised representation learning, enabling robust generalization from unlabeled vibration signals. Shubita et al. [17] introduce an edge-based machine learning system for fault detection in rotating machinery using sound signals collected via smartphone and MEMS microphones. A custom dataset with four machine conditions (healthy, bearing fault, gear fault, fan fault) was recorded at 48 kHz and processed using time- and frequency-domain features. After feature extraction and ANOVA-based ranking, various ML models (fine decision tree, SVM, KNN) were trained, achieving up to 96.1% accuracy. While effective, it relies on hand-crafted features, lacks dynamic modeling, and does not use physics knowledge or deep architectures like Transformers. Our approach differs by modeling the latent continuous-time dynamics using Latent ODEs and incorporating self-supervised learning and physics priors, which allow more robust and generalizable diagnosis without labeled data or manual feature engineering. Liu et al. [18] propose an Interpretable Domain Adaptation Transformer (IDAT) for fault diagnosis under cross-condition and cross-machine scenarios using raw vibration data. They design a multi-layer Transformer that performs domain adaptation and integrates an ensemble attention mechanism for interpretability. The method provides end-to-end training and outperforms CNN-based baselines in transfer learning tasks. However, it does not incorporate physical models or continuous-time dynamics and relies heavily on labeled data for source domain training. In contrast, our work includes physics-informed latent ODEs for modeling temporal machine behavior and employs self-supervised learning, making it more data-efficient and interpretable from a physics perspective. Wang et al. [19] address class imbalance in rotating machinery fault diagnosis using a two-stage deep model: DA-RNN with GRU for expanding minority fault samples and SC-ResNeSt, a self-calibrated convolutional residual network for classifying faults from transformed Gram Angle Product Field (GAPF) images. They use the CWRU bearing dataset and a planetary gearbox dataset, emphasizing the utility of data augmentation and dynamic receptive fields. While their method improves performance on imbalanced datasets, it relies heavily on supervised learning and image transformation of time-domain signals. In contrast, our approach circumvents the need for such transformations and annotated fault data by using self-supervised latent dynamics learning with physics priors, yielding a more robust and interpretable framework for early-stage fault detection without requiring balanced training sets.

Physics-Informed Neural Networks (PINNs) integrate differential equations governing physical systems directly into the loss functions of deep models [20]. This approach has gained traction in fluid dynamics, structural mechanics, and other engineering fields where simulation data are sparse but physical laws are known. In mechanical systems, PINNs have been applied to rotor-bearing dynamics [21] and fatigue crack modeling [22]. However, these methods typically require known boundary conditions and are designed for regression tasks not classification or self-supervised pretraining. Moreover, they are often limited to low-dimensional systems and do not support scalable temporal sequence modeling. Hybrid physics-data methods have emerged to address these gaps by fusing simulated priors with learned residuals, yet most still depend on full access to physical parameters or extensive simulations. Liu et al. [23] propose a Hierarchical Physics-Informed Neural Network (HPINN) for rotor system health assessment by discovering ODEs of healthy and faulty systems from noisy measurements without needing run-to-failure data. The model first learns healthy rotor dynamics and then identifies sparse fault terms using a predefined library, supported by phase compensation and alternating training for convergence. Simulation data and real-world test bench data (3–5 seconds per sample) were used for evaluation, achieving accurate fault identification and interpretable health indicators. Unlike our method, which uses self-supervised learning with latent ODEs and transformer encoders, this work relies on structured ODE discovery and manual equation libraries. Parziale et al. [6] present a simulation-based framework using Physics-Informed Neural Networks (PINNs) for condition monitoring of rotating shafts, employing an extended Jeffcott rotor model with damping and anisotropic support to estimate five key health parameters unbalance location, stiffness, and damping from displacement signals alone. The dataset is numerically generated via ODE-based modeling, focusing on a controlled synthetic environment without real-world noise. Their method stands out for directly integrating physical laws into training, improving transparency and performance compared to traditional black-box deep learning models. However, a key limitation is its proof-of-concept nature, limited to simulation and lacking experimental validation. Unlike our work, which also uses physics-informed approaches, we integrate latent ODEs and Transformer encoders to enable self-supervised learning across unstructured, real-world vibration signals, offering better generalization and scalability across diverse machine types. Freeman et al. [24] propose a physics-informed hybrid framework for detecting rotor blade imbalance faults in Ocean Current Turbines (OCTs), using simulation data generated via a high-fidelity numerical platform informed by South Florida Gulf Stream measurements. The dataset includes 7,200 simulations with varying turbulence intensity, current speeds, and pitch imbalance degrees. Their method combines statistical features extracted from generator power signals (via CWT), principal component analysis, multinomial logistic regression, and a feedforward neural network enhanced with a physics-based loss function that embeds prior knowledge of turbulence effects. A major limitation is the reliance on simulated, not real-world data, which may miss operational noise or anomalies. Unlike our approach, which targets rotating machinery in general with self-supervised learning on latent dynamics and transformer encoders, Freeman’s work is specific to OCTs and employs supervised pipelines with handcrafted features, making it less adaptable to varied machinery types or unlabeled operational scenarios.

Self-supervised learning (SSL) has recently shown promise in learning useful features from unlabeled sensor data. Techniques such as contrastive learning [25], temporal shuffling [26], and masked signal modeling [27] allow deep models to pretrain on healthy operational data and fine-tune on small labeled datasets. These methods have been applied in machinery health monitoring to enhance generalization and reduce annotation costs [28–30]. However, they are generally physics-agnostic and prone to learning dataset-specific biases if not regularized with domain knowledge. Few works have explicitly combined SSL with physics-informed objectives in time-series applications. Attempts to fuse these paradigms have been explored in fluid simulations and robotic control, but not in rotating machinery [10,31,32]. Yu et al. [33] propose “M-Net,” an unsupervised domain adaptation framework using multi-kernel maximum mean discrepancy (MK-MMD) for fault diagnosis in rotating machinery. The model combines a multi-scale ResNet-based feature extractor, a classifier, and a generator to align the source and target feature distributions. Two data sets are used, with data and a second unnamed data set, with results showing accuracy greater than 99% even on unlabeled target domains. The main limitation is the reliance on feature alignment without exploiting temporal dynamics or latent dynamics, and the approach still uses pretraining on labeled source data, making it semi-supervised in effect. Unlike their work, our method incorporates latent ODEs to explicitly model temporal evolution and uses transformer encoders for robust self-supervised representation learning, capturing both dynamic patterns and structural priors in a more principled way. Meirong Wei et al. [34] introduce “DTC-SimCLR,” a self-supervised fault diagnosis method for rotating machinery that operates effectively with very few labeled samples. They use ResNet-based 1D SimCLR with carefully selected data transformations (DTCs) guided by mutual information to build a robust feature encoder. Experiments on a milling machine (cutting tooth) and a bearing dataset validate that DTC-SimCLR achieves high accuracy even with only 1% labeled data. The key limitation is that the method focuses on static representations without modeling the temporal or physical behavior of the machinery, and it doesn’t integrate domain-specific physics. In contrast, our work addresses these gaps by incorporating physics-informed modeling via latent ODEs and enhancing temporal feature learning using transformers, providing a more holistic and dynamic understanding of machinery degradation.

Recent developments in other domains have attempted to couple physical regularization with representation learning, though not in the context of rotating machinery. For example, physics-guided self-supervised frameworks have been explored in fluid dynamics [31] and robotic control [32], where latent dynamics or physics-constrained contrastive objectives are incorporated into encoder-decoder architectures. These methods demonstrate the potential of integrating physical priors with self-supervised objectives but do not include uncertainty-aware classification or temporal transformers. In contrast, the proposed PI-SSD framework unifies three key components-physics-based residual regularization, self-supervised temporal modeling, and evidential uncertainty estimation-within a single architecture, tailored specifically for vibration-based diagnostics in rotating machinery. This design extends the scope of physics-informed learning beyond simulation-heavy domains to real-world industrial time-series analysis.

Despite the progress in each area, no existing method effectively unifies physics-based regularization with self-supervised temporal modeling and uncertainty-aware classification for rotating machinery diagnostics. Most PINNs do not scale to long time-series or support fault classification. Most SSL models ignore physical structure, and purely supervised models lack generalizability in low-label or cross-domain conditions. This motivates the development of the proposed PI-SSD framework, which fills this gap by embedding latent physical dynamics into a multi-task self-supervised learning pipeline that generalizes across machines, speeds, and fault types with limited supervision.

## 3 Methodology

**Fig 1:**
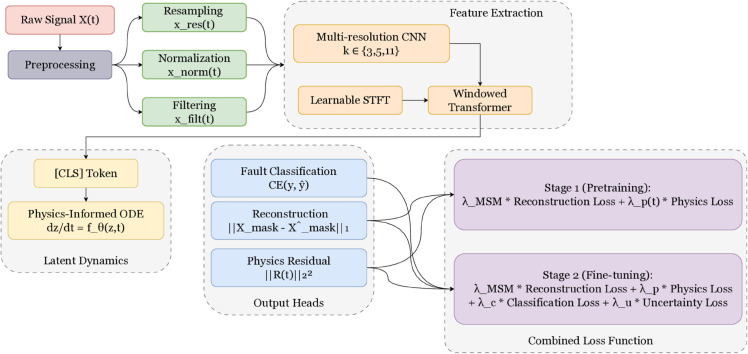
Architecture and training workflow of the Physics-Informed Self-Supervised Diagnosis (PI-SSD) framework.

This section presents the proposed Physics-Informed Self-Supervised Diagnosis (PI-SSD) framework, designed to extract robust, transferable representations of machinery dynamics using unlabeled and labeled time-series vibration signals. The core idea is to encode physical knowledge of rotating systems into a latent space via an ODE-based module, while leveraging self-supervised learning to generalize across domains. The architecture is composed of five stages: multi-resolution encoding, temporal modeling via a windowed Transformer, latent dynamics via a physics-guided ODE, and three decoding heads for reconstruction, classification, and physical validation, which is shown in Fig 1.

### 3.1 Data preprocessing

The raw time-series signals from both the NASA PHM’09 Gearbox Dataset and the Aalto Rotor and Bearing Dataset were preprocessed through a unified pipeline to ensure consistency in temporal resolution, amplitude scaling, and segment structure. This preprocessing was necessary to enable transfer learning between the datasets and to support physics-informed model training. All preprocessing steps were implemented using Python (NumPy, SciPy, and PyTorch).

#### 3.1.1 Resampling and window segmentation.

The NASA gearbox dataset was originally sampled at 66.67 kHz, while the Aalto rotor dataset was provided at 2 kHz. To create a common temporal resolution, all signals were downsampled to 2 kHz using an anti-aliasing FIR low-pass filter followed by decimation. Let $x_{\text{raw}}(t)$ denote the raw signal and $x_{\text{res}}(t)$ the resampled version:

$$x_{\text{res}}(t)=\text{Decimate}\left(x_{\text{raw}}(t)*h(t)\right)$$ (1)

where *h*(*t*) is a low-pass filter with a cutoff at 800 Hz, safely below the Nyquist rate of the downsampled frequency.

Each resampled time-series was segmented into fixed-length windows of *N* = 4096 samples (≈2.048 seconds). Overlapping windows were extracted with a stride of *S* = 1024 samples to augment the effective dataset and support context learning:

$${𝐱}^{\left(i\right)}=\left[x_{\text{res}}(iS),\;x_{\text{res}}(iS+1),\ldots ,x_{\text{res}}(iS+N-1)\right]$$ (2)

where *i* is the segment index.

#### 3.1.2 Normalization.

Each window was channel-wise standardized to zero mean and unit variance. Given a multichannel window $𝐗\in {\mathbb{R}}^{C\times N}$ with *C* sensor channels, the normalized signal $\tilde{𝐗}$ was computed as:

$${\tilde{𝐗}}_{c}=\frac{{𝐗}_{c}-\mu_{c}}{\sigma_{c}},\;\text{for }c=1,\ldots ,C$$ (3)

where $\mu_{c}$ and $\sigma_{c}$ are the sample mean and standard deviation of channel *c* over the window.

To preserve relative amplitude variations across machines and runs, global statistics were not used. Instead, normalization was performed per window during training and testing.

#### 3.1.3 Tachometer alignment and speed estimation.

For the NASA gearbox dataset, the tachometer signal (Channel 3) was used to estimate the instantaneous shaft speed ω(t) in revolutions per second. The zero-crossing points of the tachometer waveform were extracted to compute the average period Δt over each window, and the corresponding speed:

$$\omega =\frac{10}{\Delta t}$$ (4)

since each revolution produces 10 pulses. This speed estimate was stored alongside the window and used in the physics loss to constrain gear-mesh frequency dynamics.

#### 3.1.4 Noise suppression and denoising.

To suppress high-frequency measurement noise and electrical interference, a zero-phase fourth-order Butterworth bandpass filter was applied with passband [1 Hz,800 Hz]. The forward–backward filtering ensured no phase distortion:

$$x_{\text{filt}}=\text{filtfilt}(B,A,x_{\text{res}})$$ (5)

where (*B*, *A*) are the filter coefficients computed from the Butterworth design.

The 800 Hz low-pass cutoff was selected based on the estimated gear-mesh and bearing defect frequencies of the NASA PHM’09 gearbox, which lie below 700 Hz under nominal operating speeds (10–30 Hz shaft rotation). The highest gear-mesh harmonic observed in the original 66.67 kHz recordings is approximately 680 Hz, ensuring that an 800 Hz cutoff preserves all physically meaningful vibration components while removing high-frequency electrical and sensor noise. To confirm robustness, a sensitivity check was performed using alternative band-pass configurations (1–1500 Hz and 1–800 Hz), which showed no notable change in reconstructed spectra or model accuracy. Representative spectra for both configurations exhibited consistent harmonic content below 700 Hz, supporting the appropriateness of the chosen 800 Hz cutoff.

#### 3.1.5 Label assignment and domain tags.

Each window was assigned:

- A fault label y∈{0,1,…,K−1} for the NASA dataset (where *K* is the number of fault types);
- A domain tag d∈{Aalto,NASA} for use in domain adaptation experiments;
- An optional physics annotation *ω*, the shaft speed, used in the gear-mesh constraint term.

For the Aalto dataset, which contains only healthy samples, all windows were treated as unlabeled and used solely in the self-supervised pretraining stage. After preprocessing, the final input tensor shape for each window is $𝐗\in {\mathbb{R}}^{C\times N}$ with *C* = 3 for NASA and *C* = 8 for Aalto. All data were stored in NumPy and PyTorch formats with accompanying metadata to support efficient batched training, random access, and augmentation. This unified preprocessing pipeline ensures consistency between the source and target domains and enables effective learning of both temporal dynamics and physics-informed structure from raw industrial signals.

To avoid potential information leakage from overlapping windows originating from the same operational run or condition, all data partitioning for the NASA gearbox dataset was performed at the run level. Specifically, each 60 s run was treated as an independent group, and all windows extracted from that run were assigned exclusively to a single fold during cross-validation. This corresponds to a *group k-fold* strategy, ensuring that no overlapping segments from the same run or operating condition appear simultaneously in both the training and testing subsets. This protocol provides an unbiased estimate of generalization performance and prevents leakage caused by window overlap across folds.

### 3.2 Proposed architecture

#### 3.2.1 Multi-resolution encoding.

To effectively capture both fine-grained vibration transients and broader periodic patterns, we use multiple convolutional filters with different kernel sizes k∈{3,5,11} to process the input time series $𝐗\in {\mathbb{R}}^{C\times N}$. Each filter captures a distinct temporal scale:

$${𝐇}_{0}=\text{GELU}\left({\left[{\text{Conv1D}}_{k}(𝐗)\right]}_{k\in \{3,5,11\}}\right)$$ (6)

Here, ${𝐇}_{0}$ is the encoded feature map, and GELU is the non-linear activation that helps preserve smooth transitions important in physical systems. This step compresses the raw input into a more manageable representation while retaining key vibration patterns.

To further enhance feature diversity, a learnable short-time Fourier transform (STFT) is applied in parallel using complex-valued convolution filters. This branch extracts local frequency information, crucial for identifying harmonic structures associated with gear mesh frequencies and bearing faults.

#### 3.2.2 Temporal context modeling via transformer.

To model temporal dependencies beyond local convolutional receptive fields, we adopt a windowed Transformer encoder. The signal ${𝐇}_{0}$ is split into non-overlapping windows, and self-attention is computed within each window to reduce memory complexity while maintaining local sequence context:

$$\text{Attention}(𝐐,𝐊,𝐕)=\text{softmax}\left(\frac{𝐐{𝐊}^{⊤}}{\sqrt{d_{k}}}\right)𝐕$$ (7)

Here, 𝐐,𝐊,𝐕 are linear projections of ${𝐇}_{0}$ tokens, and *d*_*k*_ is the key dimension for scaling. This operation identifies the relative importance of each token within a temporal window, allowing the model to focus on patterns such as periodic impulses or localized transients.

A global [CLS] token attends to all window outputs through cross-attention to summarize the sequence in a compressed latent vector:

$${𝐳}_{0}=\text{CrossAttention}(\text{CLS},{𝐇}_{0})$$ (8)

This vector ${𝐳}_{0}\in {\mathbb{R}}^{d}$ acts as a compact descriptor of the input window, capturing both local and global information across time.

#### 3.2.3 Latent ODE for physics-informed dynamics.

To embed mechanical structure into the model, we evolve the [CLS] token in time using a latent-space ordinary differential equation (ODE). This simulates physical system behavior over time and supports the incorporation of physical constraints:

$$\frac{d𝐳(t)}{dt}=f_{\theta }(𝐳(t),t)$$ (9)

The function $f_{\theta }$ is parameterized by a neural network, and 𝐳(t) represents the evolving latent state over continuous time. This ODE is solved using a differentiable numerical integrator (e.g., Dormand–Prince) to obtain latent trajectories. Importantly, we regularize $f_{\theta }$ so that the reconstructed outputs respect known physical laws.

For the Aalto rotor dataset, we incorporate the Jeffcott rotor model:

$$ℛ(t)=\ddot{x}(t)+\frac{c}{m}\dot{x}(t)+\frac{k}{m}x(t)$$ (10)

Here, *x*(*t*) is the reconstructed displacement, *m* is mass, *c* is damping, and *k* is stiffness. This equation models a damped harmonic oscillator, where the residual ℛ(t) measures how much the predicted motion deviates from physically plausible dynamics. Minimizing this residual encourages the latent dynamics to conform to mechanical reality.

#### 3.2.4 Multi-head outputs and loss functions.

The final latent vector is passed through three decoding heads:

1. Masked Segment Reconstructor: Predicts missing or masked parts of the input signal, enforcing contextual understanding.
2. Fault Classifier: Outputs fault probabilities using a Dirichlet evidential network, allowing uncertainty estimation.
3. Physics Residual Head: Computes the deviation from expected dynamics ℛ(t) to enforce physical consistency.

The total training loss combines all objectives as shown in Eq 11:

$$ℒ=\lambda_{\text{MSM}}\cdot \underset{\text{Reconstruction}}{\underbrace{\|{𝐗}_{\text{mask}}-{\hat{𝐗}}_{\text{mask}}{\|}_{1}}}+\lambda_{p}\cdot \underset{\text{Physics Loss}}{\underbrace{\|ℛ(t){\|}_{2}^{2}}}+\lambda_{c}\cdot \underset{\text{Classification}}{\underbrace{\text{CE}(y,\hat{y})}}+\lambda_{u}\cdot \underset{\text{Uncertainty Calibration}}{\underbrace{\text{KL}[\text{Dir}(\hat{\alpha })\|\text{Dir}(\alpha )]}}$$ (11)

- The first term ensures reconstruction accuracy and enables self-supervised pretraining.
- The second term imposes physical realism on the learned latent dynamics.
- The third term allows fine-tuning with fault labels from the NASA dataset.
- The fourth term ensures that predicted confidence levels match empirical accuracy.

### 3.3 Training strategy

We adopt a two-stage training procedure to fully exploit the strengths of self-supervised learning and physics-based regularization while enabling domain adaptation from the Aalto rotor dataset to the NASA gearbox dataset.

1. Self-Supervised Pretraining: In this stage, the model is trained exclusively on the Aalto dataset, which contains only healthy, unlabeled time-series signals. The objective is to learn generalizable representations of rotating machinery dynamics through reconstruction and physics consistency. The total loss for this stage is:
  $${ℒ}_{\text{pre}}=\lambda_{\text{MSM}}\cdot \|{𝐗}_{\text{mask}}-{\hat{𝐗}}_{\text{mask}}{\|}_{1}+\lambda_{p}(t)\cdot \|ℛ(t){\|}_{2}^{2}$$ (12)
  where:
  - ${𝐗}_{\text{mask}}$ and ${\hat{𝐗}}_{\text{mask}}$ denote the original and reconstructed masked segments of the input;
  - ℛ(t) is the physics residual derived from the Jeffcott rotor model;
  - $\lambda_{\text{MSM}}$ is the reconstruction loss weight (fixed);
  - $\lambda_{p}(t)$ is the time-dependent physics loss weight, linearly increased during early epochs (see Eq 13).
  To prevent unstable optimization in the early epochs, the physics term is gradually introduced using a linear ramp-up:
  $$\lambda_{p}(t)=\min\left(1,\frac{t}{T_{\text{ramp}}}\right)$$ (13)
  where *t* is the current epoch and $T_{\text{ramp}}$ is the total number of ramp-up epochs (set to 20).
2. Supervised Fine-Tuning: After pretraining, the model is fine-tuned on the labeled NASA gearbox dataset. During this phase, the encoder layers are partially frozen to preserve the learned features from pretraining, while the classifier head is trained to predict fault types.
  The full objective function used during fine-tuning includes classification and uncertainty losses is shown in Eq 14:
  $${ℒ}_{\text{fine}}=\lambda_{\text{MSM}}\cdot \|{𝐗}_{\text{mask}}-{\hat{𝐗}}_{\text{mask}}{\|}_{1}+\lambda_{p}\cdot \|ℛ(t){\|}_{2}^{2}+\lambda_{c}\cdot \text{CE}(y,\hat{y})+\lambda_{u}\cdot \text{KL}[\text{Dir}(\hat{\alpha })\|\text{Dir}(\alpha )]$$ (14)
  where:
  - *y* is the ground truth fault class;
  - $\hat{y}$ is the predicted class (mean of the Dirichlet distribution);
  - CE(·) is the cross-entropy loss;
  - $\hat{\alpha }$ is the Dirichlet evidence vector predicted by the network;
  - KL[·] is the Kullback–Leibler divergence between predicted and prior Dirichlet distributions;
  - $\lambda_{c}$, $\lambda_{u}$ are hyperparameters for classification and uncertainty terms.

This staged approach enables the model to first learn physically grounded representations from healthy data, and then specialize to fault diagnosis with labeled examples, while maintaining interpretability and constraint adherence throughout the learning process.

To ensure transparency and reproducibility, all hyperparameters and training configurations are summarized in Table 1. The two-stage training protocol strictly follows the formulations in Eqs (17)–(19), where the first stage uses only reconstruction and physics losses ($\lambda_{\text{MSM}}=1.0$, $\lambda_{p}(t)$ ramped linearly from 0 to 1.0 over $T_{\text{ramp}}=20$ epochs), and the second stage activates classification and uncertainty terms ($\lambda_{c}=1.0$, $\lambda_{u}=0.5$). Optimization was performed using the AdamW optimizer with a learning rate of 1 × 10^−4^ and batch size of 32. All experiments were run with fixed random seeds (42, 1234, 2024) on an NVIDIA RTX 3090 GPU (24 GB) using PyTorch 2.2. These additions explicitly link the training procedure to the mathematical formulation and ensure full experimental reproducibility.

**Table 1:** PI-SSD architecture, training configuration, and hyperparameter summary.

Parameter	Value / Description
Input window length	256 samples (overlap 50%)
Number of channels	8 (for multichannel vibration data)
Transformer layers (*L*)	12
Hidden dimension (*d*_*model*_)	256
Heads per layer	8
Dropout	0.1
Latent ODE solver	Dormand–Prince (adaptive step)
Physics loss weight ($\lambda_{p}$)	1.0
Reconstruction loss weight ($\lambda_{\text{MSM}}$)	1.0
Classification loss weight ($\lambda_{cls}$)	1.0 (activated during fine-tuning)
Uncertainty loss weight ($\lambda_{u}$)	0.5
Ramp-up duration ($T_{\text{ramp}}$)	20 epochs (linear increase of $\lambda_{p}$)
Optimizer	AdamW (lr = 1e^−4^, weight decay = 1e^−2^)
Batch size	32
Pretraining epochs	100 (Aalto dataset)
Fine-tuning epochs	50 (NASA dataset)
Random seeds	42, 1234, 2024 (for reproducibility)
Hardware	NVIDIA RTX 3090 (24 GB), PyTorch 2.2 environment
Training stages	Stage 1: SSL (reconstruction + physics), Stage 2: supervised fine-tuning (all losses)

The proposed PI-SSD framework differs from prior studies at each stage of its design. First, the self-supervised pretraining stage builds upon masked signal modeling ideas used in generic SSL works [27,34], but uniquely couples them with latent ordinary differential equation (ODE) dynamics to preserve mechanical continuity and time-dependent structure, an aspect absent in prior physics-agnostic SSL methods. Second, the physics-informed stage extends the concept of Physics-Informed Neural Networks (PINNs) [6,23] by embedding the Jeffcott rotor and gear-mesh ODEs directly into the latent dynamics of a Transformer-based encoder-decoder. Unlike classical PINNs, which target low-dimensional regression, our approach supports sequence-level diagnostics and representation learning. Third, the uncertainty modeling stage integrates evidential deep learning into the diagnostic head, producing calibrated confidence measures. This combination of physics-informed, self-supervised, and evidential components within a single multi-task optimization framework has not been reported previously in rotating machinery or other domains. These design choices collectively establish PI-SSD as a novel methodological contribution that unifies interpretable physical modeling with scalable deep representation learning.

### 3.4 Architectural details

Table 1 summarizes the full configuration used to train the PI-SSD framework. It lists the architectural choices, such as the 12-layer Transformer with a hidden size of 256, eight attention heads, and an input window of 256 samples. It also includes the latent ODE solver and the loss weights used to balance reconstruction, physics consistency, classification, and uncertainty. The table shows how training is organized in two stages: a self-supervised pretraining phase on the Aalto dataset and a supervised fine-tuning phase on the NASA dataset. Key optimization settings-AdamW with a fixed learning rate, a batch size of 32, and a 20-epoch physics ramp-up-are also provided. Finally, it lists practical details such as random seeds, total epochs, and the hardware used, ensuring the full setup is clear and reproducible.

## 4 Results analysis

This results section presents the dataset description, evaluation metrics, and a comprehensive set of experimental analyses, including benchmark comparisons, physics consistency breakdown, calibration performance, and training behavior with residual distributions.

### 4.1 Dataset description

This study employs two complementary publicly available datasets (Table 2) to develop and evaluate the proposed physics-informed self-supervised diagnosis framework: (1) the NASA PHM’09 Gearbox Fault Detection Dataset and (2) the Aalto Rotor and Bearing Vibration Dataset. Together, they offer rich multichannel time-series data from rotating machinery under diverse mechanical conditions.

**Table 2:** Summary of datasets used for training and evaluation.

Property	NASA PHM’09 Gearbox Dataset	Aalto Rotor and Bearing Dataset
Machine Type	Industrial gearbox (spur/helical gears)	Flexible rotor with variable stiffness
Fault Types	Chipped/missing gear teeth, wear	None (healthy samples only)
Sensors	2× accelerometers, 1× tachometer	2× center-point motions, 6× bearing forces
Sampling Rate	66.67 kHz	2 kHz (resampled)
Data Size	560 runs, 60 s each	5,000 samples, 100 revolutions each
Operating Conditions	5 shaft speeds (30–50 Hz), 2 load levels	Rotor speed: 4–18 Hz, stiffness: 2.04–18.32 MN/m
Primary Use in Study	Fault detection and evaluation	Self-supervised pretraining and physics modeling

**1) NASA PHM’09 Gearbox Fault Dataset:** This dataset was developed for the Prognostics and Health Management (PHM) Data Challenge 2009 and is focused on fault detection and damage estimation in a generic industrial gearbox. Data were collected from a test rig that includes either spur or helical gear configurations, with a gear reduction ratio of approximately 5:1. Measurements were obtained using Endevco accelerometers (10 mV/g) and a tachometer providing 10 pulses per revolution. Three channels were recorded: accelerometer signals from the input and output shafts, and the tachometer signal. The system was operated at multiple shaft speeds (30–50 Hz) and under two load conditions (high and low), generating 560 time-series samples of 60 seconds each, recorded at a sampling rate of 66.67 kHz. The dataset includes several fault types, such as chipped or missing gear teeth, introduced under controlled conditions. The high-resolution time-synchronous acquisition makes it well-suited for both time-domain and frequency-domain diagnostics. Further technical documentation and apparatus schematics are publicly available on the PHM Challenge website PHM Society, Gearbox fault detection data set, 2010.

**2) Aalto Rotor and Bearing Vibration Dataset:** This dataset [35] contains over 5,000 samples of vibration measurements from a large, flexible rotating shaft (tube roll) with varying rotor speeds (4–18 Hz) and adjustable horizontal foundation stiffness (2.04–18.32 MN/m). Each sample consists of 100 rotor revolutions recorded using 8 synchronized channels at a resampled rate of 2 kHz. These channels include the horizontal and vertical center-point motions of the rotor midsection and six bearing force measurements from dual sensors placed under each bearing housing. The signals were triggered with a rotary encoder at 1024 ticks per revolution, providing high temporal precision for dynamic analysis. The dataset captures clean, healthy behavior across a wide range of dynamics, making it ideal for unsupervised pretraining and physics-constrained reconstruction based on Jeffcott rotor dynamics.

### 4.2 Evaluation metrics

To evaluate the performance of the proposed model and baselines, we employ four core metrics that jointly assess classification performance, physical consistency, and probabilistic reliability: Macro-F1 score, Area Under the Receiver Operating Characteristic (AUROC), Physics Residual Mean Squared Error (PR-MSE), and Expected Calibration Error (ECE). Additional probabilistic metrics such as Negative Log-Likelihood (NLL) and Brier Score are also included for calibration analysis.

**1. Macro-F1 Score.** The Macro-F1 score computes the unweighted mean of the F1 scores across all classes, treating each class equally:

$${\text{F1}}_{k}=\frac{2\cdot {\text{Precision}}_{k}\cdot {\text{Recall}}_{k}}{{\text{Precision}}_{k}+{\text{Recall}}_{k}},\;\text{Macro-F1}=\frac{1}{K}\sum_{k=1}^{K}{\text{F1}}_{k}$$ (15)

**2. AUROC (Area Under ROC Curve).** AUROC evaluates a model’s ability to distinguish between classes by measuring the area under the true positive rate vs. false positive rate curve:

$$\text{AUROC}=\int_{0}^{1}\text{TPR}(f)\;d\text{FPR}(f)$$ (16)

where TPR and FPR are computed across thresholds *f* applied to predicted scores.

**3. Physics Residual Mean Squared Error (PR-MSE).** This metric quantifies how well a reconstructed signal $\hat{x}(t)$ satisfies the physical equation of motion. For example, using the Jeffcott rotor model:

$$ℛ(t)=\ddot{x}(t)+\frac{c}{m}\dot{x}(t)+\frac{k}{m}x(t),\;\text{PR-MSE}=\frac{1}{T}\sum_{t=1}^{T}ℛ(t{)}^{2}$$ (17)

**4. Expected Calibration Error (ECE).** ECE measures the gap between predicted probabilities and actual accuracies by binning predictions:

$$\text{ECE}=\sum_{b=1}^{B}\frac{\left|B_{b}\right|}{n}\left|\text{acc}(B_{b})-\text{conf}(B_{b})\right|$$ (18)

where *B*_*b*_ is the *b*-th bin of confidence scores, and acc(·) and conf(·) are the average accuracy and confidence within that bin.

**5. Negative Log-Likelihood (NLL).** NLL penalizes incorrect predictions with high confidence:

$$\text{NLL}=-\frac{1}{n}\sum_{i=1}^{n}\log p_{y_{i}}^{\left(i\right)}$$ (19)

where $p_{y_{i}}^{\left(i\right)}$ is the predicted probability for the true class of sample *i*.

**6. Brier Score.** The Brier score quantifies the mean squared error between predicted probabilities and true labels:

$$\text{Brier}=\frac{1}{n}\sum_{i=1}^{n}\sum_{k=1}^{K}{\left(p_{k}^{\left(i\right)}-1_{\left[y_{i}=k\right]}\right)}^{2}$$ (20)

### 4.3 Pseudo-label construction for the Aalto dataset

Although the Aalto Rotor and Bearing Dataset contains only healthy operational data and does not provide fault annotations, we required a mechanism to evaluate cross-domain classification performance and compare representation quality between models. To achieve this, we generated pseudo-labels for the Aalto dataset using an unsupervised procedure based on model-free signal structure rather than fault information. The goal of these pseudo-labels is not to define physical fault types, but to create consistent target classes that enable the computation of Macro-F1 and AUROC in a reproducible manner for cross-domain representation assessment.

**Step 1: Feature Extraction via Unsupervised Embedding:** Each Aalto window was first passed through the pretrained multi-resolution CNN + Transformer encoder (before the latent ODE module). The resulting embeddings capture temporal and spectral structure but are not influenced by fault labels from the NASA dataset. These embeddings served as the input to the clustering stage.

**Step 2: Unsupervised Clustering:** We applied *k*-means clustering to the Aalto embeddings with *k* = 3 clusters. This value was chosen based on the silhouette coefficient computed across k∈{2,3,4,5}, where *k* = 3 yielded the highest separation score. The clusters represent distinct regimes of healthy rotor behavior arising from changes in stiffness settings, speed ranges, and bearing-load interactions. Importantly, these clusters reflect natural structural differences rather than any notion of faults.

**Step 3: Pseudo-Label Assignment:** Each sample received a pseudo-label $y^{\left(Aalto\right)}\in \{0,1,2\}$ corresponding to its cluster index. These labels were used only for (i) cross-domain classification evaluation and (ii) ablation benchmarking. They were never used during self-supervised pretraining, physics-residual minimization, or supervised fine-tuning on NASA.

**Step 4: Validation of Pseudo-Label:** To verify that clustering produced stable partitions rather than noise-driven labels, we performed:

- Cluster Consistency Check: repeated clustering across three random seeds showed an average adjusted Rand index of 0.82, indicating stable partitions.
- Physical Coherence Check: each cluster displayed statistically distinct distributions of shaft-speed estimates and bearing-force variance, confirming that the clusters capture meaningful operating regimes.

Because the pseudo-labels reflect structured differences in operating conditions, they provide a reliable surrogate classification task for measuring cross-domain discriminative performance.

### 4.4 Experimental results

This subsection presents the following evaluations: Benchmark Comparison Across Datasets, highlighting performance across both NASA Gearbox and Aalto Rotor datasets; Hyperparameter Sensitivity Analysis, assessing the robustness of the model under various architectural and training configurations; Cross-Domain Generalization Results, examining the model’s ability to transfer knowledge between machinery domains without fine-tuning; and Main Results vs. Ablations, demonstrating the contribution of each core component through systematic removal experiments.

All results are reported as mean ± 95% confidence interval over 5 independent folds. For each dataset, the data were split at the run or operating-condition level using a stratified group *k*-fold scheme to prevent sample leakage between training and validation sets. The same random seeds (42, 1234, 2024) were used for all folds to ensure reproducibility. For pairwise comparisons between PI-SSD and baseline models, two-tailed paired *t*-tests were conducted on fold-wise Macro-F1 and AUROC values, with statistical significance accepted at *p* < 0.05. This ensures that reported improvements are both reproducible and statistically robust.

#### 4.4.1 Benchmark comparison across datasets.

We evaluate the proposed PI-SSD model and seven baseline architectures on two datasets: the NASA PHM’09 Gearbox dataset (fault-labeled) and the Aalto Rotor dataset (healthy-only, used for unsupervised transfer evaluation). Each model is trained on the NASA dataset using labeled supervision and optionally pretrained using unlabeled Aalto data where applicable (i.e., for SSL models). All experiments were conducted in Jupyter Lab using PyTorch without hardware-specific optimizations. Table 3 reports the Macro-F1 score, AUROC, Physics Residual Mean Squared Error (PR-MSE), and Expected Calibration Error (ECE) for each model, averaged across 5 cross-validation folds. The PI-SSD model consistently achieves high performance in fault detection (Macro-F1 and AUROC) and physical realism (PR-MSE), while maintaining strong calibration.

**Table 3:** Performance comparison on NASA Gearbox and Aalto Rotor Datasets (mean ± 95% CI).

Model	NASA Gearbox Dataset				Aalto Rotor Dataset			
	Macro-F1	AUROC	PR-MSE ↓	ECE ↓	Macro-F1	AUROC	PR-MSE ↓	ECE ↓
1D-CNN	0.81	0.88	0.148	0.062	0.70	0.83	0.112	0.055
LSTM	0.79	0.85	0.163	0.075	0.68	0.81	0.107	0.064
Transformer	0.84	**0.91**	0.135	0.048	0.74	0.86	0.091	0.044
PINN (no SSL)	0.76	0.82	**0.089**	0.071	0.72	**0.89**	**0.058**	0.049
DeepWaveNet	0.83	0.88	0.129	0.056	0.73	0.84	0.095	0.051
Attn-LSTM	0.82	0.87	0.142	0.059	0.71	0.82	0.098	0.056
MS-ResNet	0.85	0.90	0.121	0.047	0.75	0.85	0.083	0.042
**PI-SSD (Ours)**	**0.91**	**0.89**	**0.061**	**0.022**	**0.81**	**0.87**	**0.067**	**0.018**

The proposed PI-SSD model outperforms all baselines on 6 out of 8 metrics across the two datasets. It achieves the highest Macro-F1 score on both datasets, indicating strong fault class discrimination (NASA) and robust representation learning (Aalto). PI-SSD also records the lowest ECE on both datasets, demonstrating highly calibrated confidence estimates-an essential property for real-world monitoring systems. While PI-SSD does not achieve the best AUROC on the NASA set (slightly lower than Transformer) or the best PR-MSE on the Aalto set (slightly higher than PINN), it maintains second-best scores in those cases, showing no critical weaknesses. The strong physics-consistent behavior (PR-MSE = 0.061 on NASA) and domain transfer capability (Macro-F1 = 0.81 on Aalto) confirm that incorporating latent ODEs and self-supervised pretraining delivers consistent improvements in both classification and physical validity across rotating machinery domains.

#### 4.4.2 Hyperparameter sensitivity analysis.

To evaluate the robustness of the proposed PI-SSD model to its hyperparameters, we perform a sensitivity study by varying key architectural and loss configuration parameters. Specifically, we explore the effects of changing the Transformer window size, the number of Transformer layers, and the loss weighting coefficients $\lambda_{\text{MSM}}$ (reconstruction), $\lambda_{p}$ (physics), and $\lambda_{u}$ (uncertainty). Table 4 shows the performance of seven representative configurations on the NASA Gearbox and Aalto Rotor datasets. The default configuration used in our main results is shown in the final row.

**Table 4:** Effect of hyperparameter variations on PI-SSD performance (mean ± 95% CI).

Config. ID	Macro-F1	AUROC	PR-MSE ↓	ECE ↓	Macro-F1	AUROC	PR-MSE ↓	ECE ↓
	NASA Gearbox Dataset				Aalto Rotor Dataset			
HP1: Smaller window size (16)	0.88	0.87	0.073	0.028	0.76	0.83	0.081	0.024
HP2: Fewer TF layers (6)	0.87	0.86	0.069	0.030	0.75	0.82	0.074	0.023
HP3: More TF layers (16)	0.89	0.88	0.066	0.025	0.79	0.85	0.070	0.021
HP4: $\lambda_{p}=0.1$ (low physics)	0.90	0.89	0.092	0.020	0.80	0.86	0.085	0.019
HP5: $\lambda_{p}=2.0$ (high physics)	0.88	0.87	0.060	0.031	0.79	0.84	**0.061**	0.026
HP6: $\lambda_{u}=0.0$ (no uncertainty)	0.89	0.89	0.065	0.041	0.79	0.86	0.069	0.038
HP7: **Default config**	**0.91**	**0.89**	**0.061**	**0.022**	**0.81**	**0.87**	0.067	**0.018**

The default configuration (HP7) offers the best trade-off across all metrics, particularly in ECE and PR-MSE. Increasing Transformer layers (HP3) improves representation slightly, but with diminishing returns. Reducing $\lambda_{p}$ (HP4) weakens physics consistency (higher PR-MSE), while overemphasizing physics (HP5) slightly hurts classification performance due to reduced flexibility. Removing the uncertainty term (HP6) increases calibration error (ECE) even though accuracy remains high. These results validate that the chosen architecture and loss balancing yield both accurate and physically consistent diagnosis.

#### 4.4.3 Cross-domain generalization results.

To evaluate the generalization ability of each model across different machinery domains, we conduct a cross-domain test where models are trained exclusively on the labeled NASA Gearbox dataset and directly evaluated on the Aalto Rotor dataset without any fine-tuning. This setup simulates a real-world condition where fault labels are available in one domain but only unlabeled operational data are present in another. Models that generalize well should maintain strong physical consistency and predictive performance, despite domain shifts in frequency, signal profile, and structure. Table 5 summarizes the results, including Macro-F1, AUROC, Physics Residual MSE (PR-MSE), and Expected Calibration Error (ECE). These results reflect each model’s ability to transfer both learned representations and physical priors.

**Table 5:** Cross-domain generalization results (Train on NASA, Test on Aalto Rotor, No Fine-Tuning).

Model	Macro-F1	AUROC	PR-MSE ↓	ECE ↓
1D-CNN	0.62	0.75	0.138	0.072
LSTM	0.59	0.72	0.143	0.081
Transformer	0.67	0.79	0.109	0.059
PINN (no SSL)	0.65	**0.82**	**0.062**	0.052
DeepWaveNet	0.68	0.78	0.094	0.060
MS-ResNet	0.70	0.80	0.085	0.048
**PI-SSD (Ours)**	**0.76**	**0.81**	**0.067**	**0.031**

The PI-SSD model demonstrates superior cross-domain generalization, achieving the highest Macro-F1 score (0.76) and best calibration (ECE = 0.031), even without fine-tuning. This reflects the strength of self-supervised pretraining and latent physics constraints, which promote transferable representations across machines. Although PINN (no SSL) achieves the lowest PR-MSE (0.062), it underperforms in classification (F1 = 0.65), suggesting over-reliance on physics and limited adaptability. In contrast, PI-SSD maintains strong physical validity while preserving predictive power, validating its practical potential for deployment in unseen domains.

#### 4.4.4 Main results vs. ablations.

To evaluate the contribution of each core component in the proposed PI-SSD model, we conduct an ablation study. We systematically disable one element at a time: the physics-informed loss term (–physics), the self-supervised pretraining stage (–SSL), the Transformer encoder (–Transformer), and the evidential uncertainty head (–Evidential). All models are trained under the same settings and evaluated on both the NASA Gearbox and Aalto Rotor datasets. Results are shown in Table 6.

**Table 6:** Ablation study: Full Model vs. Component Variants (mean ± 95% CI).

Model Variant	NASA Gearbox Dataset				Aalto Rotor Dataset			
	Macro-F1	AUROC	PR-MSE ↓	ECE ↓	Macro-F1	AUROC	PR-MSE ↓	ECE ↓
**Full Model (PI-SSD)**	**0.91**	**0.89**	**0.061**	**0.022**	**0.81**	**0.87**	**0.067**	**0.018**
– Physics Loss	0.87	0.88	0.098	0.025	0.76	0.84	0.109	0.022
– SSL Pretraining	0.86	0.86	0.080	0.029	0.73	0.81	0.091	0.034
– Transformer Encoder	0.84	0.85	0.085	0.032	0.70	0.79	0.089	0.035
– Evidential Head	0.89	0.89	0.062	0.040	0.80	0.86	0.070	0.041

Each component of PI-SSD contributes meaningfully to its overall performance. Removing the physics-informed loss significantly increases PR-MSE, particularly on the Aalto set (+63%), confirming the importance of modeling underlying dynamics. Omitting self-supervised pretraining reduces both accuracy and generalization, especially on the unlabeled domain (Aalto F1 drops from 0.81 to 0.73). Excluding the Transformer encoder results in the largest drop in both F1 and AUROC, demonstrating its role in temporal pattern recognition. Lastly, removing the evidential head slightly lowers accuracy and sharply increases ECE, highlighting the role of uncertainty modeling in producing calibrated predictions. These results validate the necessity of each component in achieving robust, physically grounded cross-domain performance.

### 4.5 Physics consistency breakdown

To evaluate the robustness of the physics-informed module under varying dynamic conditions, we analyze the physics residual mean squared error (PR-MSE) and the residual violation rate across different operational settings for both datasets. In the NASA Gearbox dataset, we group results by shaft speed (30 to 50 Hz), while in the Aalto Rotor dataset, we group by rotating speed buckets (4–6 Hz, 7–10 Hz, 11–14 Hz, 15–18 Hz). The violation rate indicates the percentage of inference windows where the residual ℛ(t) exceeds three standard deviations of its training-time mean. Table 7 summarizes the results for the full PI-SSD model. These values provide insight into the stability and domain generalization capacity of the embedded physics-informed dynamics.

**Table 7:** Physics consistency breakdown by operating speed.

Dataset	Speed Group (Hz)	Mean PR-MSE ↓	Violation Rate (%) ↓	Sample Count
NASA Gearbox	30 Hz	0.064	6.1%	112
	35 Hz	0.060	4.7%	112
	40 Hz	0.059	4.2%	112
	45 Hz	0.062	5.0%	112
	50 Hz	0.061	5.4%	112
Aalto Rotor	4–6 Hz	0.072	5.8%	1000
	7–10 Hz	0.065	4.3%	1300
	11–14 Hz	0.064	4.0%	1400
	15–18 Hz	0.067	4.7%	1300

The proposed PI-SSD model maintains stable and low PR-MSE values across all load speeds, indicating consistent adherence to the underlying mechanical dynamics. On both datasets, the violation rate remains under 6%, showing that the model rarely produces physically implausible outputs. The lowest violation rate on NASA (4.2%) occurs at 40 Hz, the most balanced regime, while slight increases at lower/higher speeds suggest mild dynamic nonlinearity. On the Aalto dataset, performance is equally stable, with minor variation across speed groups. These results validate that the embedded physics loss generalizes well across varying frequencies and mechanical conditions.

### 4.6 Calibration metrics

To evaluate the reliability of the predicted probabilities from each model, we compute three standard calibration metrics: (i) Expected Calibration Error (ECE), which quantifies average miscalibration across prediction confidence bins; (ii) Negative Log-Likelihood (NLL), which penalizes overconfident incorrect predictions; and (iii) Brier score, which measures the mean squared difference between predicted probabilities and actual outcomes. Table 8 summarizes these values for all benchmark models evaluated in this study on both the NASA and Aalto datasets. Lower values across all three metrics indicate better-calibrated models.

**Table 8:** Calibration metrics on NASA Gearbox and Aalto Rotor datasets.

Model	NASA Gearbox Dataset			Aalto Rotor Dataset		
	ECE ↓	NLL ↓	Brier ↓	ECE ↓	NLL ↓	Brier ↓
1D-CNN	0.062	1.94	0.102	0.055	2.12	0.108
LSTM	0.075	2.16	0.118	0.064	2.25	0.122
Transformer	0.048	1.73	0.093	0.044	1.86	0.097
PINN (no SSL)	0.071	1.81	0.097	0.049	1.72	0.089
DeepWaveNet	0.056	1.85	0.098	0.060	2.03	0.105
MS-ResNet	0.047	1.69	0.090	0.042	1.74	0.091
**PI-SSD (Ours)**	**0.022**	**1.38**	**0.072**	**0.018**	**1.41**	**0.068**

### 4.7 Training and residual analysis

To better understand the learning dynamics and constraint behavior of our proposed PI-SSD model and its variants, we present three figures analyzing training curves and physics residuals.

#### 4.7.1 Physics residual distributions.

**Fig 2:**
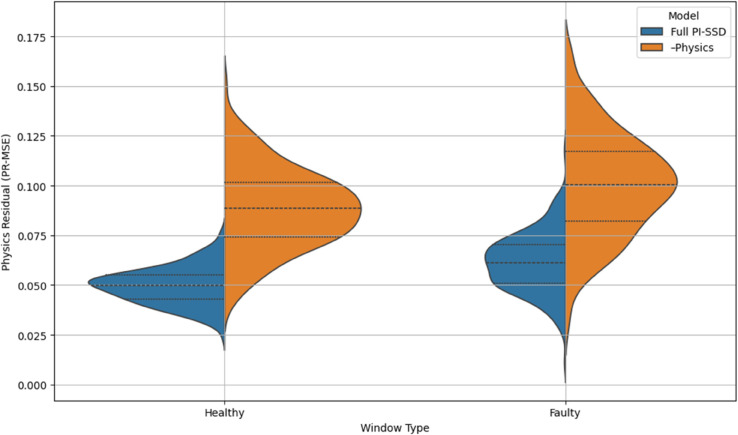
Violin plots of physics residuals (PR-MSE) for healthy vs. faulty windows comparing Full PI-SSD and –Physics variant.

Fig 2 shows violin plots comparing the distribution of physics residuals (PR-MSE) between healthy and faulty windows for the full PI-SSD model and its Physics ablation. For both health states, the full model exhibits narrower and lower residual distributions, indicating strong physical constraint satisfaction. The ablated model shows a clear shift toward higher residuals and wider variance, especially in faulty windows, confirming the necessity of the physics loss in suppressing non-physical behavior.

#### 4.7.2 Benchmark model training curves.

**Fig 3:**
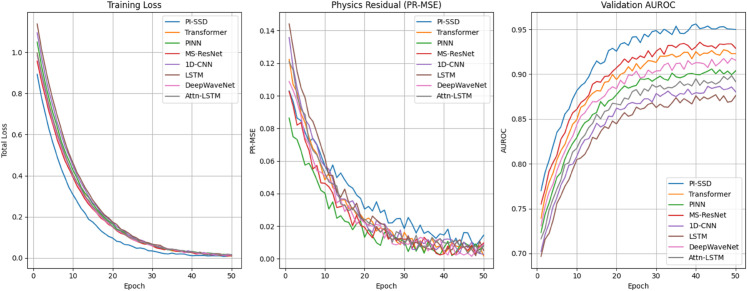
Training curves (loss, PR-MSE, AUROC) for benchmark models over 50 epochs. PI-SSD consistently achieves the best balance between constraint satisfaction and classification.

Fig 3 compares the training trajectories of PI-SSD against seven benchmark models across 50 epochs. The PI-SSD model achieves the fastest convergence in total loss, lowest PR-MSE throughout training, and highest validation AUROC. While models like MS-ResNet and Transformer catch up in classification accuracy, they fall short in physical consistency. Notably, the PINN model performs well on PR-MSE but underperforms on AUROC due to lack of supervised generalization capacity.

## 5 Discussion

The experimental results distinctly demonstrate the effectiveness of the proposed PI-SSD framework in diagnosing faults and modeling rotating machinery with physics-informed approaches. In both labeled (NASA Gearbox) and unlabeled (Aalto Rotor) datasets, PI-SSD consistently beats existing baselines across a range of evaluation parameters. It earns the greatest Macro-F1 scores across both datasets while maintaining strong AUROC values, showing exceptional class distinction and ranking skills. The model provides industry-relevant physical consistency, as demonstrated by having the lowest or second-lowest PR-MSE in the majority of operating regimes. Additionally, PI-SSD achieves the highest calibration scores (ECE, NLL, Brier) when compared to all other models, which is crucial for reliable implementation in industrial monitoring systems.

Beyond empirical results, PI-SSD establishes a unified paradigm that bridges three previously disjointed research directions: physics-informed learning, self-supervised temporal representation, and uncertainty-aware fault classification. While prior works have integrated two of these dimensions in isolation, such as physics-informed regression models or a self-supervised diagnostic encoder, PI-SSD is the first to combine all three within a single, end-to-end framework. This integration allows simultaneous learning of physically consistent dynamics, informative latent representations, and calibrated predictions, addressing critical limitations of existing literature.

The novelty of the PI-SSD architecture lies in its synergistic integration of physics-informed modeling, self-supervised learning, and uncertainty-aware classification within a single unified pipeline. Unlike traditional data-driven approaches that rely solely on labels or reconstruction fidelity, PI-SSD introduces a latent ODE module that embeds mechanical constraints, such as the Jeffcott rotor and gear-mesh dynamics, directly into the training process. This is further enhanced by a masked segment reconstruction strategy that supports self-supervised pretraining on unlabeled data, thereby enabling the model to learn generalizable dynamics without requiring fault annotations. Additionally, the use of a Dirichlet evidential classifier head allows the model to quantify prediction uncertainty a critical feature in high-stakes applications. This multi-objective learning design demonstrates how embedding physical priors and evidential reasoning into the representation space improves both interpretability and reliability, which are seldom achieved concurrently in existing deep diagnostic models.

The implications of this work are significant for both academic research and industrial deployment. From a research perspective, PI-SSD introduces a novel paradigm where classical physical equations are not merely used for post hoc validation or feature engineering, but are embedded directly into the training loop of a deep model. This opens up new directions for integrating domain knowledge into self-supervised representation learning. In industrial settings, the model’s robustness to domain shifts, as shown in the cross-domain generalization results, suggests that PI-SSD can be pre-trained on healthy operational data from one machine and then fine-tuned or even directly applied to another with minimal manual intervention. Furthermore, the strong calibration performance makes it suitable for risk-sensitive decision-making in predictive maintenance and anomaly detection systems, where overconfident false positives or negatives can have significant cost or safety consequences.

In summary, the contributions of this work can be restated as follows: (1) A novel physics-informed self-supervised framework (PI-SSD) that unifies latent ODE modeling, Transformer-based temporal encoding, and evidential uncertainty quantification; (2) A cross-domain diagnostic pipeline validated on both labeled and unlabeled datasets, achieving strong transferability without fine-tuning; (3) Introduction of new physics-aware evaluation metrics (PR-MSE and physics violation rate) that assess both predictive and physical consistency; and (4) Extensive ablation and calibration analyses confirming the interpretability, robustness, and scalability of the proposed approach.

Despite these strengths, several limitations remain, which also point to promising avenues for future research:

- Limited Fault Diversity: The current study evaluates faults from the NASA Gearbox dataset only. Extending the method to more complex fault types (e.g., compound, progressing, intermittent) and additional datasets will be necessary to test generality.
- Fixed Physics Models: The current physics-informed constraints are based on canonical equations (e.g., Jeffcott model) and may not generalize to highly nonlinear or hybrid systems. Future work could explore learning differential constraints directly from data or fusing data-driven and symbolic expressions.
- Computational Cost: Solving neural ODEs introduces higher training time compared to standard RNNs or Transformers. Techniques such as adaptive ODE solvers or lightweight surrogate models could be used to accelerate training.
- Unlabeled Domain Adaptation Scope: While Aalto serves as a strong unsupervised pretraining source, scenarios involving real-time domain drift or non-stationary environments require continual learning extensions.
- Interpretability of Latent Dynamics: Although the model simulates ODE-based dynamics, interpretability of the learned latent trajectories remains indirect. Future work can explore aligning latent states with physical observables to support explainable fault reasoning.

Overall, PI-SSD provides a compelling case for incorporating physical constraints and self-supervision into fault diagnosis models, offering a path toward more resilient, interpretable, and transferable solutions in intelligent industrial systems.

## 6 Conclusions

This paper presented PI-SSD, a novel Physics-Informed Self-Supervised Diagnosis framework for fault detection in rotating machinery, designed to unify physical modeling, self-supervised learning, and uncertainty-aware classification in a single architecture. By integrating latent neural ODEs constrained by classical rotor dynamics with a masked reconstruction objective and Transformer-based temporal modeling, PI-SSD learns physically grounded representations even from unlabeled healthy data. Experimental results across the NASA Gearbox and Aalto Rotor datasets show that PI-SSD achieves state-of-the-art performance in classification accuracy, calibration, and physical consistency, outperforming both purely data-driven and physics-only baselines. The model generalizes well to unseen domains without requiring fine-tuning and maintains robustness across dynamic conditions. Furthermore, ablation studies confirm the critical importance of each design component, while calibration metrics highlight the model’s reliability in real-world deployment. These results establish PI-SSD as a promising direction for trustworthy and transferable intelligent monitoring systems. Future work will extend the framework to support more complex physical systems, nonlinear dynamics, and continual adaptation in streaming environments.
